# Improvement of the Antioxidant and Antitumor Activities of Benzimidazole-Chitosan Quaternary Ammonium Salt on Drug Delivery Nanogels

**DOI:** 10.3390/md22010040

**Published:** 2024-01-11

**Authors:** Bing Ma, Qing Li, Jingjing Zhang, Yingqi Mi, Wenqiang Tan, Zhanyong Guo

**Affiliations:** 1 Key Laboratory of Coastal Biology and Bioresource Utilization, Yantai Institute of Coastal Zone Research, Chinese Academy of Sciences, Yantai 264003, China; bingma@yic.ac.cn (B.M.); qli@yic.ac.cn (Q.L.); jingjingzhang@yic.ac.cn (J.Z.); yqmi@yic.ac.cn (Y.M.); wqtan@yic.ac.cn (W.T.); 2 University of Chinese Academy of Sciences, Beijing 100049, China; 3 Center for Ocean Mega-Science, Chinese Academy of Sciences, 7 Nanhai Road, Qingdao 266071, China

**Keywords:** benzimidazole-chitosan quaternary ammonium salt, acidic-responsive nanogels, antioxidant activity, antitumor activity

## Abstract

The present study focused on the design and preparation of acid-responsive benzimidazole-chitosan quaternary ammonium salt (BIMIXHAC) nanogels for a controlled, slow-release of Doxorubicin HCl (DOX.HCl). The BIMIXHAC was crosslinked with sodium tripolyphosphate (TPP) using the ion crosslinking method. The method resulted in nanogels with low polydispersity index, small particle size, and positive zeta potential values, indicating the good stability of the nanogels. Compared to hydroxypropyl trimethyl ammonium chloride chitosan-Doxorubicin HCl-sodium tripolyphosphate (HACC-D-TPP) nanogel, the benzimidazole-chitosan quaternary ammonium salt-Doxorubicin HCl-sodium tripolyphosphate (BIMIXHAC-D-TPP) nanogel show higher drug encapsulation efficiency and loading capacity (BIMIXHAC-D-TPP 93.17 ± 0.27% and 31.17 ± 0.09%), with acid-responsive release profiles and accelerated release in vitro. The hydroxypropyl trimethyl ammonium chloride chitosan-sodium tripolyphosphate (HACC-TPP), and benzimidazole-chitosan quaternary ammonium salt-sodium tripolyphosphate (BIMIXHAC-TPP) nanogels demonstrated favorable antioxidant capability. The assay of cell viability, measured by the MTT assay, revealed that nanogels led to a significant reduction in the cell viability of two cancer cells: the human lung adenocarcinoma epithelial cell line (A549) and the human breast cancer cell line (MCF-7). Furthermore, the BIMIXHAC-D-TPP nanogel was 2.96 times less toxic than DOX.HCl to the mouse fibroblast cell line (L929). It was indicated that the BIMIXHAC-based nanogel with enhanced antioxidant and antitumor activities and acidic-responsive release could serve as a potential nanocarrier.

## 1. Introduction

Clinical chemotherapy drugs have been frequently employed for tumor treatment in past years and currently. These drugs have the ability to enhance the survival rate of cancer patients [1,2,3]. Nonetheless, they are burdened with lethal side effects including the rapid emergence of resistant cells and toxic effects, which call for the development of a new approach [4,5]. An example of a classic chemotherapy drug that is frequently employed in the treatment of breast cancer and lung cancer is Doxorubicin HCl (DOX.HCl) [6,7,8]. However, DOX.HCl does not possess the ability to specifically target tumor tissues and can still result in significant adverse effects on healthy tissues and organs [9,10,11,12]. Due to its strong attraction to myocardial cells, DOX.HCl has the potential to induce myocardial cell fibrosis and apoptosis by producing oxygen-free radicals [13,14,15]. What strategies can be utilized to counteract these adverse effects? The development of nanocarriers with enhanced tumor accumulation capacity and antioxidant activity is crucial for delivering DOX.HCl, while eliminating free radicals, and thereby enhancing the therapeutic outcomes.

Nanocarriers are commonly employed to dissolve drugs, enhance their bioavailability, and safely improve their effectiveness by controlling the speed, timing, and location of drug release while introducing therapeutic substances into the body [16,17]. Nanogels have been extensively studied due to their flexibility, good mechanical stability, biocompatibility, and internal network structures. This makes them a promising approach for achieving a specific delivery region and timing of administration for maximum effectiveness in treating cancer [18,19,20,21,22,23,24,25,26]. Nanogels, a prominent category of soft materials with three-dimensional (3D) networks, are exceptional carriers due to their high drug-loading capacity, ease of functionalization, and responsiveness to stimuli through the introduction of various crosslinking agents and monomers [27,28]. Nanogels exhibit great stability in harsh physiological conditions and offer numerous benefits in cancer therapy because of their cross-linked structures. Moreover, these responsive nanogels can release effective substances by modifying themselves within tumor tissues or cells, according to the extracellular or intracellular environment. Nanogels are exceptionally sensitive to diverse stimuli owing to their distinctive 3D structures. Acidosis, high levels of reactive oxygen species (ROS), redox reactions, hypoxia, special enzyme overexpression, and temperature are commonly employed as triggers to initiate structural changes in nanogels [19,29,30,31,32,33]. The tumor microenvironment is characterized by acidosis, which is both a typical pathological and physiological feature. This acidic state is typically attributed to the specific metabolic pathway of tumor cells, known as the “Warburg effect”, where even when there is enough oxygen, tumor cells generate significant quantities of lactate through anaerobic glycolysis. Growing evidence suggests that acidosis plays a role in tumor metastasis, drug resistance, angiogenesis, and immune evasion [34,35,36,37]. In summary, the distinctive characteristics of the tumor microenvironment offer a logical and appealing chance to create acid-responsive nanogels made of polysaccharides, which can concentrate specifically in tumor cells. Polysaccharide nanogels are highly promising as carriers for delivering bioactive substances.

In recent years, there has been a significant focus on polysaccharide nanogels due to their remarkable modifiability, biocompatibility, and biodegradability. Researchers have successfully created nanogels containing acidic-responsive functional groups in order to achieve tumor treatments that can be triggered by changes in an acid environment [38,39]. Acidic-responsive nanogels have the ability to accumulate in tumor tissues by exploiting the enhanced permeability and retention effect (EPR). Additionally, once they are taken up by endocytosis pathways, these nanogels can selectively release drugs in endosomes or lysosomes where the pH is controlled. Several biodegradable polysaccharides, including chitosan and chitosan quaternary ammonium salts, have been extensively researched for the development of site-specific nanocarriers. These systems enable the targeted delivery of therapeutic agents exclusively to tumor sites [40,41,42]. Polysaccharide carriers exhibit great potential in the field of nanocarriers, offering beneficial biological properties such as antioxidant and antitumor effects. Within the realm of polysaccharide-derived polymers, chitosan and chitosan quaternary ammonium salts are extensively utilized due to their ability to construct anticancer nanocarriers, as well as their biocompatibility, biodegradability, and non-toxicity [43,44].

In nature, chitosan stands out as the sole positive charge polymer. Common positive charge polymers, similar to the chitosan structure, are modified quaternary ammonium salts, such as *N*-trimethyl chitosan, hydroxypropyl trimethyl ammonium chloride chitosan (HACC), and HACC derivatives [45]. Our previous study showcased the positive qualities of HACC and HACC derivatives, including their biocompatibility, nontoxicity, biodegradability, antioxidant properties, bacteriostatic effect, and antitumor activity [46]. Furthermore, HACC and its derivatives have gained popularity as carrier materials for nanogels, making them promising candidates for nanogel preparation. Mi et al. successfully prepared HACC and HACC grafting folic acid and used sodium tripolyphosphate (TPP) for ionic gelation to create nanogels that served as carriers for doxorubicin. These nanogels exhibited acidic responsiveness and ensured consistent drug release, offering notable advantages [47]. Nowadays, there is still a requirement for the advancement of new nanogels that exhibit favorable properties such as biocompatibility, biodegradability, and the ability to be easily customized.

**Scheme 1:**
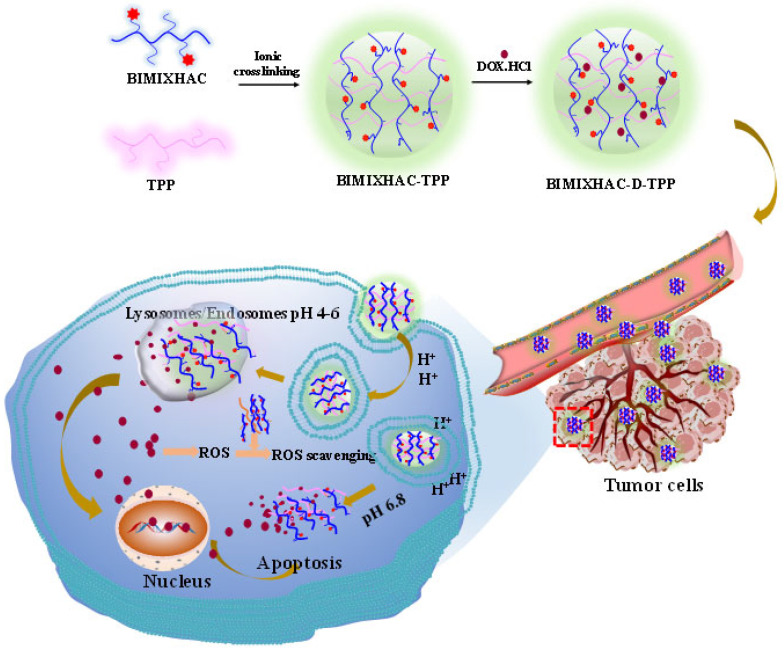
DOX.HCl release and scavenging of reactive oxygen species (ROS) of BIMIXHAC-D-TPP nanogel under acidic conditions.

In order to decrease the cardiotoxicity of DOX.HCl and enhance its accumulation at tumor sites, we developed active targeting nanogels (as shown in Scheme 1). In this study, we present the results of our investigation on acidic-responsive nanogels derived from hydroxypropyl trimethyl ammonium chloride chitosan (HACC), which exhibit responsiveness to acidic conditions. To achieve this goal, we adopted a grafting strategy to incorporate 2-benzimidazolecarboxylic acid (BIM), an acidic-responsive molecule, into HACC, resulting in a modified HACC known as BIMIXHAC. Afterward, we employed the ion crosslinking technique and used TPP as the polyanionic crosslinking molecules in order to generate blank nanogels called HACC-TPP and BIMIXHAC-TPP. These blank nanogels were then used strategically to precisely control the release of the model drug DOX.HCl. The release behavior of HACC-D-TPP and BIMIXHAC-D-TPP nanogels under different pH values (7.4, 6.5, and 5.0) was studied, and their DPPH radical scavenging activity and superoxide anion radical scavenging activity was tested. Additionally, the added benzimidazole rings were found to reduce the free radicals produced by DOX.HCl, thus enhancing its antitumor effect and minimizing its cardiotoxicity. Finally, the antitumor activity against the human lung adenocarcinoma epithelial cell line (A549) and human breast cancer cell line (MCF-7) was evaluated in vitro using the MTT assay. To confirm their potential for targeted antitumor treatment, our research focused on investigating the nanogels formed through ionic crosslinking in terms of the particle size, sustained release, antioxidant activity, biocompatibility, acidic responsiveness, and antitumor effect.

## 2. Results

### 2.1. Chemical Synthesis and Characterization

#### 2.1.1. FT-IR Spectra

**Figure 1:**
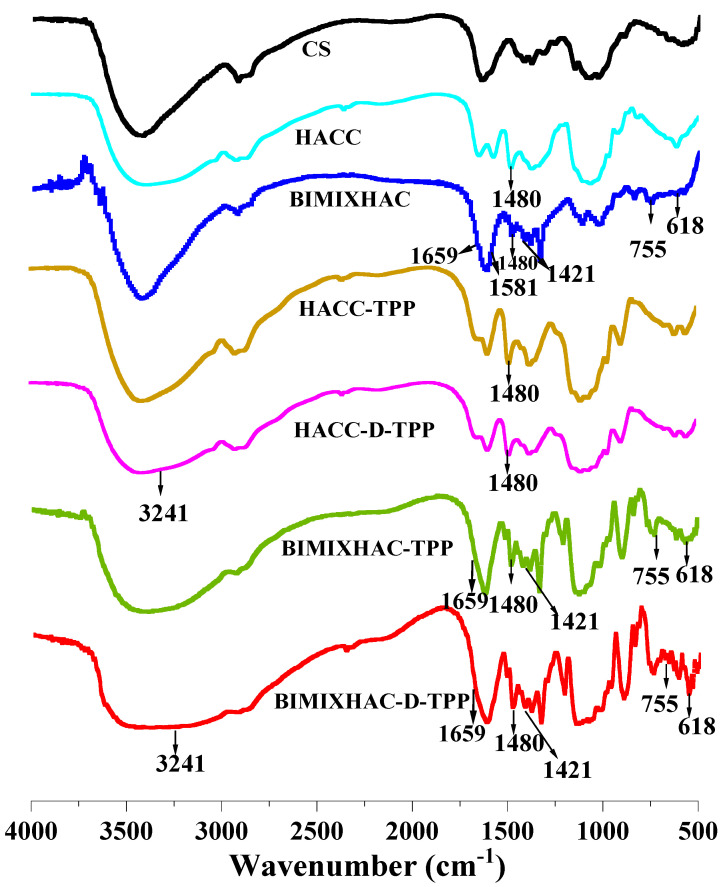
FT-IR spectra of HACC, BIMIXHAC, and all the nanogels.

The FT-IR spectra of the synthesized chitosan derivative and the prepared nanogels are shown in Figure 1. For chitosan, the major peaks appeared at 3419 cm^−1^, 1607 cm^−1^, 1155 cm^−1,^ and 1031 cm^−1^ which were the stretching vibrations of N–H and O–H, –NH_2_, and C–O at the C6 and C3 positions [48]. There was a sharp peak at 1480 cm^−1^ which confirmed the –N^+^(CH_3_)_3_ was grafted in chitosan. The spectrum data indicate the successful synthesis of HACC [49,50,51]. The new peaks at 1659 cm^−1^ and 1581 cm^−1^ were the stretching vibrations of C=C and the C=N in the imidazole ring, respectively. The new peaks at 1418 cm^−1^, 1071 cm^−1^, and 755 cm^−1^ were the stretching vibrations of the C–H in the imidazole ring. The stretching vibration of the C–N in the imidazole ring peak appeared at 618 cm^−1^ [52,53]. The above data indicate the successful synthesis of BIMIXHAC.

The FT-IR spectra of HACC-TPP, HACC-D-TPP, BIMIXHAC-TPP, and BIMIXHAC-D-TPP nanogels are shown in Figure 1. In the spectra of HACC-TPP, HACC-D-TPP, BIMIXHAC-TPP, and BIMIXHAC-D-TPP, there were characteristic quaternary ammonium salt peaks at 1480 cm^−1^. TPP interacted with the cationic groups of HACC and BIMIXHAC to form ammonium phosphate, whose characteristic peaks were 1565 and 1375 cm^−1^, respectively. Compared with the blank nanogels without DOX.HCl, the DOX.HCl-loaded nanogels showed a significant broadening peak at 3241 cm^−1^, indicating that DOX.HCl was successfully loaded onto the blank nanogels [54]. The above data proved the successful preparation of all the nanogels. 

##### H NMR Spectroscopy

**Figure 2:**
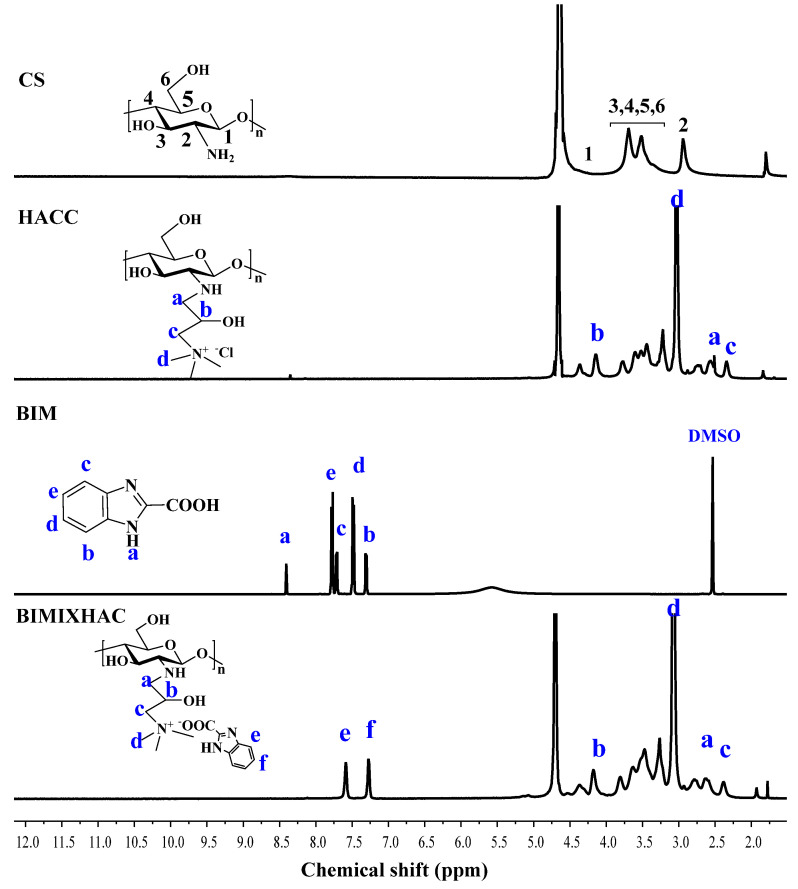
^1^H NMR spectra of CS, HACC, BIM, and BIMIXHAC.

The chemical shifts of chitosan were at 4.5 ppm (the H1 of chitosan), 1.9 ppm (the protons of H2), in the range of 3.3–4.0 ppm (the protons of H3–H6), and 3.0 ppm (the protons of glucosamine unit). The spectra of chitosan, HACC, BIM, and BIMIXHAC are shown in Figure 2. A sharp proton signal peak appeared at 3.10 ppm, which demonstrated that the –N^+^–(CH_3_)_3_ groups were grafted onto HACC and BIMIXHAC [55]. Meanwhile, the peaks appearing at 4.19, 2.64, and 2.43 ppm were attributed to the CH_2_ (c), CH (b), and CH_2_ (a) of HACC, respectively. As for BIM, the chemical shifts appearing at 8.35, 7.61, 7.58, 7.35, and 7.28 ppm were attributed to the NH (a), CH (b), CH (c), CH (d), and CH (e), respectively. In the spectrum of BIMIXHAC, the peaks at 7.58 and 7.28 ppm were the protons of CH (e), and CH (g) in the benzimidazole ring [56], respectively. The above chemical shift values proved that the benzimidazole ring was grafted onto HACC. Obviously, the above analysis further verified that the HACC and BIMIXHAC were successfully synthesized. Furthermore, the DS of HACC and BIMIXHAC were 81.50% and 34.50%, respectively.

### 2.2. Characterization of Nanogels

#### 2.2.1. Zeta Potential, Particle Size, Polydispersity Index (PDI), and Stability of Nanogels

Table 1 displays the determination of zeta potential, particle size, and PDI for all the nanogels. Firstly, compared to the blank nanogels (HACC-TPP and BIMIXHAC-TPP), the size of the DOX.HCl-loaded nanogels (HACC-D-TPP and BIMIXHAC-D-TPP) experienced different degrees of increase. The particle size of nanocarriers is a crucial factor. Drug molecules that are smaller than 100 nm can effectively enter the vascular epithelial cells of normal tissues, thereby losing their specificity towards tumor tissues. On the other hand, molecules larger than 500 nm are highly susceptible to experiencing a first-pass effect or being swiftly eliminated by liver drug enzymes. Consequently, they lack stability and possess low drug formability [57]. The nanogels have a diameter ranging from 132.21 to 242.78 nm, making them suitable for being taken up by endocytosis and the reticuloendothelial system, and accumulating in tumor cells passively through the enhanced permeability and retention effect (EPR effect) [58]. The zeta potential is an important parameter for assessing the stability of nanogels. Typically, a higher absolute value of the zeta potential indicates a more stable particle dispersion system. In Table 1, all nanogels exhibited a positive zeta potential, which ranged from +18.12 ± 0.06 to +20.97 ± 0.17 mV, respectively. The PDI indexes for all nanogels were below 30%, which indicated a higher level of uniform distribution in the solutions.

**Figure 3:**
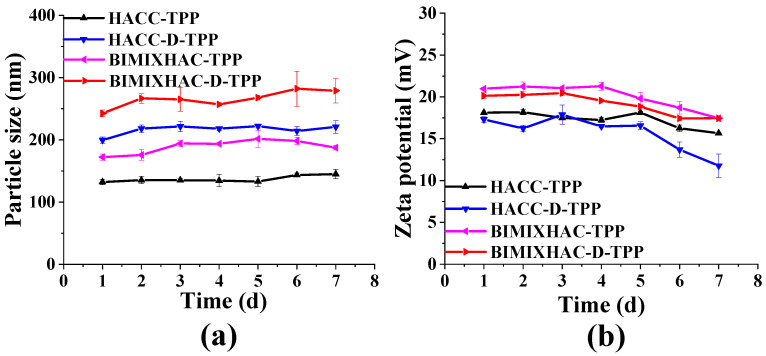
Particle size stability (**a**) and zeta potential stability (**b**) of HACC-TPP, HACC-D-TPP, BIMIXHAC-TPP, and BIMIXHAC-D-TPP nanogels.

Figure 3 presents the findings from studying the stability of all the nanogels. It is observed that the particle size of these nanogels remained fairly constant during the 7 days storage period, indicating their superior stability. The increase in particle size could be attributed to the aggregation of particles, while the decrease in conductivity could be attributed to both the continuous aggregation of the nanogels and the decreasing concentration of cationic polymers in the solution over time. In short, these nanogels exhibited a spherical morphology, a positive zeta potential, and excellent stability, making them suitable carriers for DOX.HCl in the delivery system and providing protection against its degradation.

#### 2.2.2. Morphology of Nanogels

**Figure 4:**
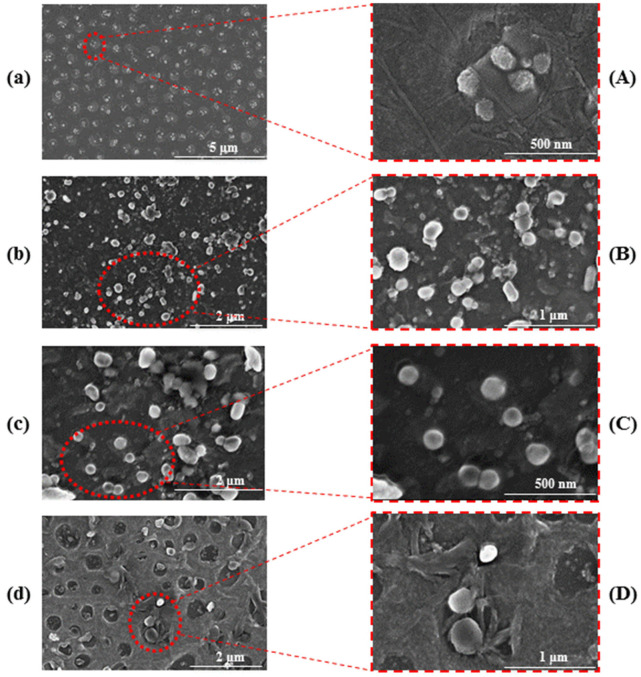
The SEM of all the nanogels. (**a**,**A**) The image of HACC-TPP; (**b**,**B**) the image of HACC-D-TPP; (**c**,**C**) the image of BIMIXHAC-TPP; (**d**,**D**) the image of BIMIXHAC-D-TPP; (**a**–**d**) and local magnification image corresponding to (**A**–**D**), respectively.

**Figure 5:**
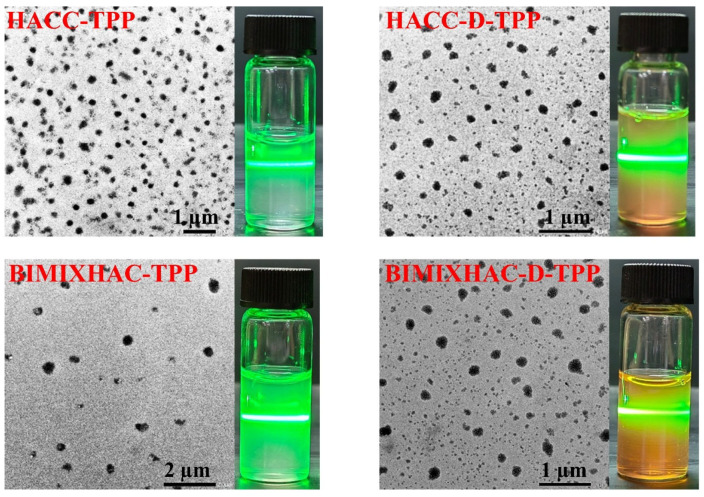
The TEM and the dispersion stability of all the nanogels.

Figure 4 and Figure 5 illustrate that the morphology of the various nanogels consisted of nearly spherical particles, which further confirmed that HACC and BIMIXHAC created nanogels using the ion crosslinking method. The particle size of all the nanogels, observed through SEM and TEM, was slightly smaller than the size determined by the dynamic light scattering method. This difference may be attributed to the fact that the nanogel solutions used for SEM and TEM imaging were subjected to air drying conditions, resulting in the contraction of the nanogels. The particle size of the bland nanogels was expected to be approximately 30–90 nm less than that of the DOX.HCl-loaded nanogels. This was potentially influenced by the efficiency of entrapment and loading.

#### 2.2.3. Drug Entrapped Efficiency and Loading Efficiency of Nanogels

Table 1 displays the drug entrapped efficiency (EE) and loading efficiency (LE) of the HACC-D-TPP and BIMIXHAC-D-TPP nanogels. These nanogels demonstrated a higher EE and LE, exceeding 90% and 26%, respectively. BIMIXHAC-D-TPP exhibited the highest encapsulation rate and loading rate (93.17% and 31.17%), respectively, surpassing the previously reported value [59]. DOX.HCl-loaded nanogels based on BIMIXHAC are superior to those based on HACC in terms of the EE and LE. This is primarily because BIMIXHAC possesses a benzimidazole structure, which allows for the formation of numerous structural cavities that can effectively encapsulate DOX.HCl. Moreover, the choice of crosslinking agent significantly impacts the capacity for drug entrapment and loading. The drug can be well protected and have a longer stability and shelf life when the EE is higher and the drug is properly sealed. Meeting clinical needs becomes easier when the drug loading capacity is larger. In order to achieve optimal drug efficacy and stability, it is necessary to consider the encapsulation efficiency and drug loading capacity during the design and preparation process of drug formulations.

### 2.3. In Vitro Drug Release

**Figure 6:**
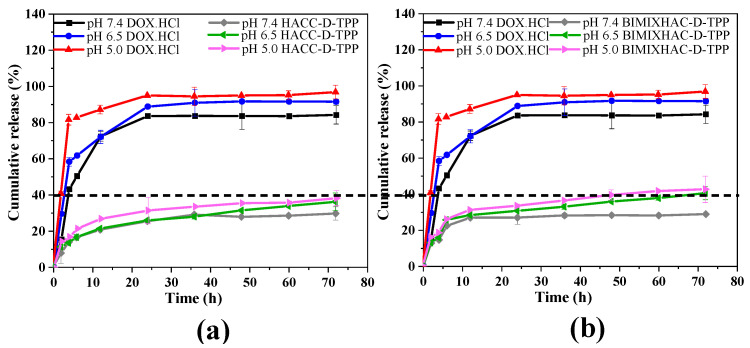
In vitro DOX.HCl release of DOX.HCl-loaded nanogels under different pH conditions: (**a**) HACC-D-TPP and (**b**) BIMIXHAC-D-TPP.

The aim of this assay was to determine the acidic-responsive property of the DOX.HCl-loaded nanogels, which closely mimic the acidic nature of the tumor tissue microenvironment. The DOX.HCl release of nanogels loaded with DOX.HCl (HACC-D-TPP and BIMIXHAC-D-TPP) was carried out using buffer solutions at different pH values (7.4, 6.5, 5.0). The findings are illustrated in Figure 6. Initially, the quick release of free DOX.HCl occurred within the first 4 h, resulting in a cumulative release percentage of 81.63% at pH 5.0. Within 24 h, free DOX.HCl demonstrated slow release behavior, allowing for a more thorough release. Decreasing the pH slightly enhanced the release rate and increased the amount released. In comparison, the release rate of the DOX.HCl-loaded nanogels were considerably slower than that of free DOX.HCl, with a peak of 94.85% achieved within 24 h. Furthermore, BIMIXHAC-D-TPP exhibited a higher release amount and release rate compared to HACC-D-TPP under the same conditions and acidic levels. Within the initial 4 h at pH 5.0, the cumulative release percentage of BIMIXHAC-D-TPP experienced a rapid increase, with a release index that measured 18.47%. This accelerated release during the first 4 h can be attributed to the diffusion of DOX.HCl through the surface and nearby pores of the DOX.HCl-loaded nanogels. The release rate of HACC-D-TPP and BIMIXHAC-D-TPP was 38.18% and 42.46% at pH 5.0 and at 72 h, respectively. The reason for the higher release rate of a DOX.HCl-loaded nanogel based on BIMIXHAC, compared to a DOX.HCl-loaded nanogel based on HACC, was attributed to the existence of benzimidazole structures. The benzimidazole rings have the ability to obtain protons in acidic surroundings, which increases their solubility in water and results in the contraction of the structures of the DOX.HCl-loaded nanogels. As a result, this facilitates the gradual release of DOX.HCl. Thirdly, there was no notable variance in the DOX.HCl release rate of all nanogels at a pH level of 7.4. This indicates that when the pH value is normal in the body, the DOX.HCl-loaded nanogels are capable of releasing a maximum of 28.00% (HACC-D-TPP) and 29.53% (BIMIXHAC-D-TPP) in regular tissue cells, respectively. Consequently, unlike free DOX.HCl, the DOX.HCl-loaded nanogels do not excessively release a large quantity of drugs in regular cells, thereby averting toxicity to normal tissues. The primary release of the DOX.HCl-loaded nanogels occur within the acidic tumor microenvironment, which has a lower pH value. As a result, this accelerates the release of DOX.HCl. Hence, this suggests that the HACC-D-TPP and BIMIXHAC-D-TPP nanogels exhibit acidic sensitivity. Moreover, the DOX.HCl release rate from BIMIXHAC-based nanogels was superior to that from HACC-based nanogels.

### 2.4. Antioxidant Activity

Due to the lack of a catalase in heart tissue and a weak antioxidant capacity, DOX.HCl can generate a significant amount of reactive oxygen species (ROS), leading to the indiscriminate movement of these radicals within the body, directly causing DNA strand breakage. Consequently, the mitochondrial DNA of cardiomyocytes is highly susceptible to damage by ROS [8,60,61,62,63]. Hence, it is crucial to address the issue of eliminating excessive ROS produced by DOX.HCl during tumor cell eradication. We opted for two oxygen free radicals, namely DPPH radicals and superoxide radicals, to assess the antioxidant efficacy of CS, HACC, BIMIXHAC, HACC-TPP nanogel, and BIMIXHAC-TPP nanogels.

**Figure 7:**
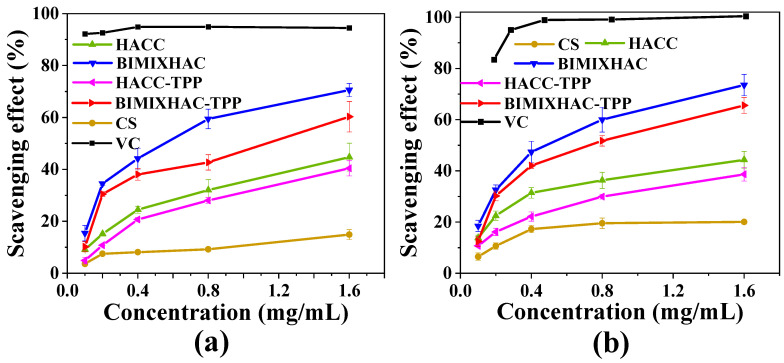
DPPH radical scavenging ability (**a**) and superoxide anion radical scavenging ability (**b**) of CS, HACC, BIMIXHAC, and HACC-TPP nanogels and the BIMIXHAC-TPP nanogel.

The DPPH radical scavenging ability of CS, HACC, BIMIXHAC, and HACC-TPP nanogels, and the BIMIXHAC-TPP nanogel is shown in Figure 7a. Initially, the nanogels based on BIMIXHAC exhibited a stronger antioxidant capacity compared to those based on HACC. The scavenging ability of HACC-TPP and BIMIXHAC-TPP on DPPH radicals was 40.34% and 60.27%, respectively. These results are mainly attributed to the fact that the scavenging ability of BIMIXHAC on DPPH radicals is significantly better than that of HACC. Our previous study reported the potential antioxidant mechanism of BIMIXHAC [46]. A hydrogen atom can be donated by the nitrogen located at the 1 position on the benzimidazole ring, causing the elimination of the single electron on the free radical, and leading to the formation of a nitrogen-centered free radical. Through resonance, the produced nitrogen-centered free radical spreads out and stabilizes the single electron of the free radical, thereby exhibiting an antioxidant effect comparable to that of a hydroxyl group. Figure 7b graphically illustrates the consistent trend of superoxide anion radical scavenging activity among different nanogels. The ranking of superoxide anion clearance, at the concentration of 1.6 mg/mL, was as follows: BIMIXHAC-TPP > HACC-TPP. It was evident that the nanogels containing BIMIXHAC exhibited a superior scavenging ability compared to those containing HACC. Furthermore, BIMIXHAC-TPP demonstrated an ideal ability to eliminate superoxide anion radicals, with a rate of 65.56%. The antioxidant capacity of HACC-TPP and BIMIXHAC-TPP was slightly weaker than that of the corresponding HACC and BIMIXHAC because a small amount of cross-linking agent TPP was added to HACC and BIMIXHAC.

Based on the analysis shown in Figure 7, it can be concluded that nanogels based on BIMIXHAC demonstrated significantly better antioxidant activity compared to nanogels based on HACC. This superiority can be attributed to the ability of the benzimidazole structure to trap electrons and donate hydrogen, which plays a crucial role in scavenging free radicals. Furthermore, the benzimidazole ring serves as an effective structure for nanocarriers. Therefore, the BIMIXHAC-TPP utilized in this study holds promise as nanocarriers with antioxidant properties.

### 2.5. Hemolysis Analysis

**Figure 8:**
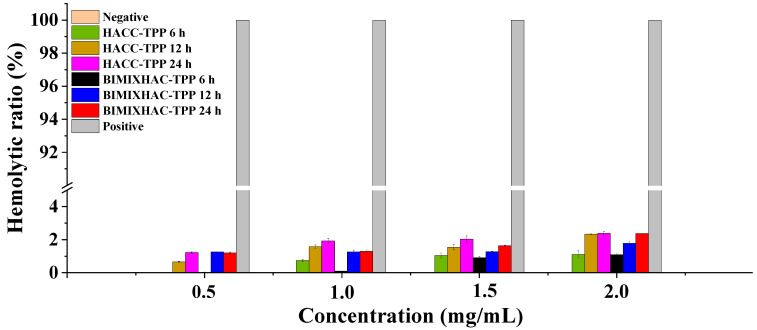
The hemolytic ratio of HACC-TPP and BIMIXHAC-TPP nanogels at 6, 12, and 24 h.

As nanocarriers, hemolytic activity is an important factor in assessing biocompatibility to determine the biosafety of the application [64]. The hemocompatibility of blank nanogels (HACC-TPP and BIMIXHAC-TPP) was investigated by hemolysis experiments. The hemolytic activity of blank nanogels at different concentrations (0.5, 1.0, 1.5, and 2.0 mg/mL) was determined at 6, 12, and 24 h, as in Figure 8. Initially, the hemolysis rate of the blank nanogels showed an increasing trend with increasing concentrations. When the concentration of the blank nanogel reached 2.0 mg/mL at 24 h, the hemolysis rates of HACC-TPP and BIMIXHAC-TPP reached 2.39% and 2.40% respectively, which were always lower than 5%. Apparently, the nanogels of HACC-TPP and BIMIXHAC-TPP can be safely used as nanocarriers.

### 2.6. Cytotoxicity Assay

**Figure 9:**
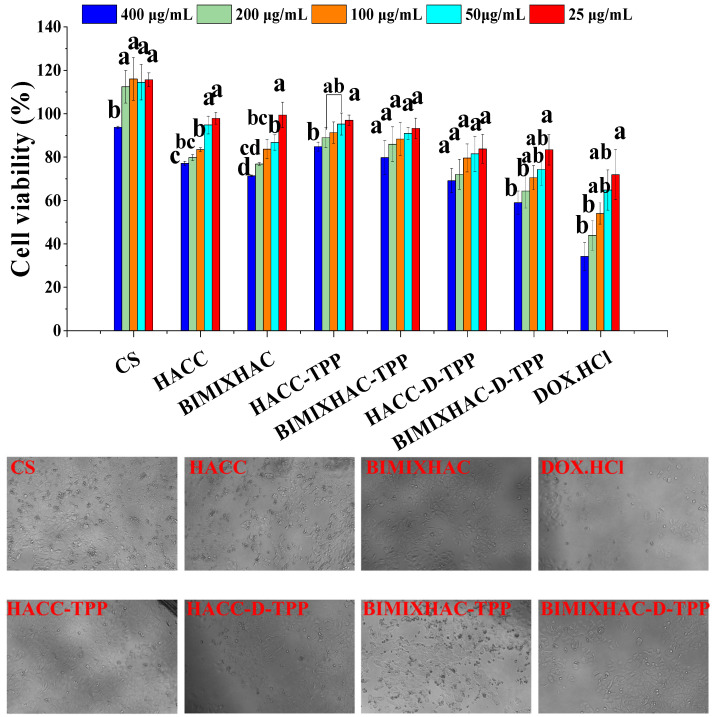
The cytotoxicity of all the nanogels on MCF-7 cells. The letters a–d are described as average value ± SD (n = 3, *p* < 0.05).

To evaluate the anticancer efficacy of HACC-TPP, BIMIXHAC-TPP, HACC-D-TPP, and BIMIXHAC-D-TPP, the cytotoxicity of free DOX.HCl and all the nanogels on MCF-7, A549, and L929 cells were observed in vitro for 48 h. Notably, a higher antitumor activity was associated with lower cell viability in the samples. As shown in Figure 9, it is clear that the cell activity of all the nanogels was significantly higher than that of free DOX.HCl. The cell viability of MCF-7 cells was 69.23%, 59.12%, and 34.34% after 48 h of HACC-D-TPP, BIMIXHAC-D-TPP, and DOX.HCl, at the concentration of 400 μg/mL, respectively. Because these nanogels were acid-responsive and their drug release rates changed at acidic pH, the cytotoxicity of HACC-D-TPP and BIMIXHAC-D-TPP was lower than that of DOX.HCl. Their relative drug release rates were 38.18%, 42.46%, and 92.78% at pH = 5.0, respectively, according to the prior in vitro sustained release experiment. The drug release rate had an impact on the cytotoxicity of the DOX.HCl-loaded nanogels. The strength of the cytotoxicity increases with the DOX.HCl release rate. The sensitivity of benzimidazole rings to acids is thought to be the cause of the BIMIXHAC-D-TPP nanogel potent cytotoxicity. The benzimidazole rings take protons at pH 6.5 and 5.0, changing the solubility of the nanogel and triggering the collapse of their spherical forms, which releases DOX.HCl from the spherical structures.

**Figure 10:**
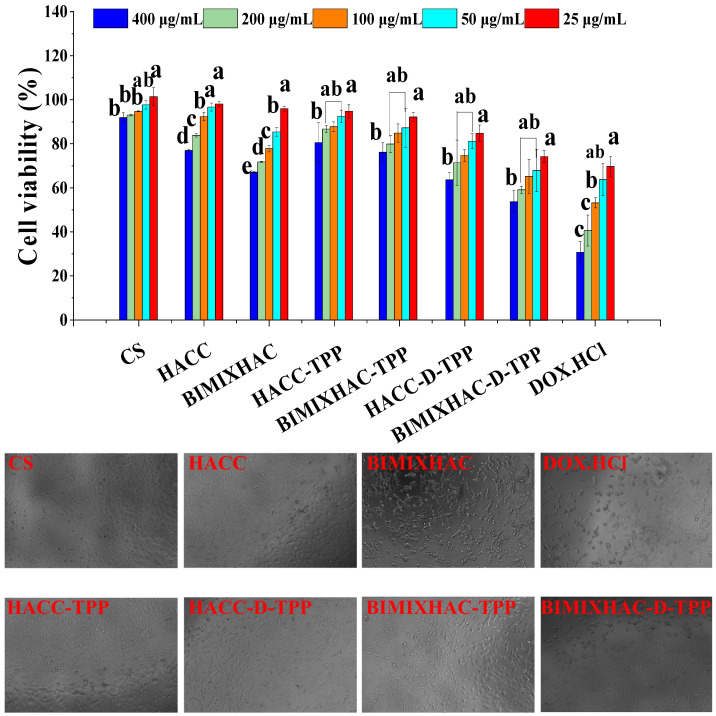
The cytotoxicity of all the nanogels on A549 cells. The letters a–d are described as average value ± SD (n = 3, *p* < 0.05).

The biocompatibility of all the nanogels in Figure 10 was evaluated using the cytotoxicity assay against A549 cells for 48 h. At a concentration of 400 μg/mL, it was obvious that the two blank nanogels (HACC-TPP and BIMIXHAC-TPP) were cytotoxic to A549 cells. They minimally varied in cytotoxicity at the same concentration. When the blank nanogels were stacked with DOX.HCl, the cytotoxicity of HACC-D-TPP was weaker than that of BIMIXHAC-D-TPP, showing that a BIMIXHAC-based nanogel was more poisonous to A549 cells. The cytotoxicity of HACC-D-TPP and BIMIXHAC-D-TPP was marginally weaker than that of DOX.HCl, in light of the fact that DOX.HCl-loaded nanogels were acid-responsive at tumor locales, which was confirmed by DOX.HCl release tests in vitro. It is essential that the poisonousness of DOX.HCl to normal L929 cells is much higher than DOX.HCl-loaded nanogels. This is precisely the exact thing we anticipate since normal cells have a slightly alkaline pH of 7.4. The nanogels we designed, the HACC-D-TPP and BIMIXHAC-D-TPP, do not respond at normal pH, and, therefore, the DOX.HCl wrapped inside the nanogels would not be delivered, better safeguarding normal cells.

**Figure 11:**
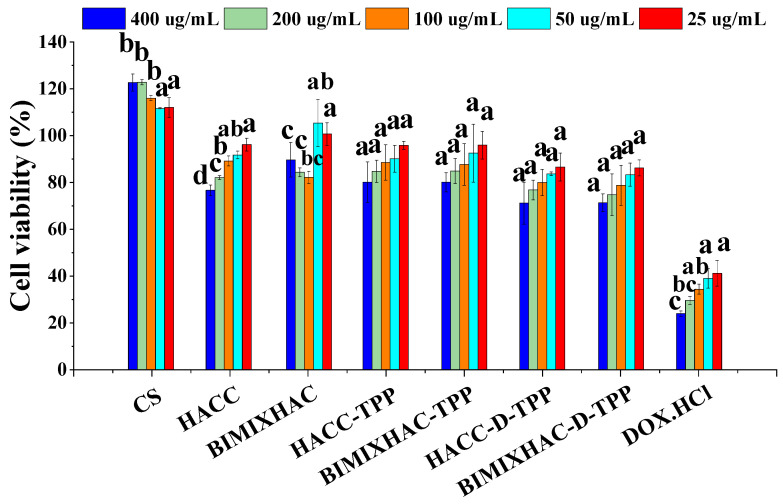
The cytotoxicity of all the nanogels on L929 cells. The letters a–d are described as average value ± SD (n = 3, *p* < 0.05).

Meanwhile, a cytotoxicity assay was performed on L929 cells for 48 h to assess the biocompatibility of two blank nanogels and two DOX.HCl-loaded nanogels, as shown in Figure 11. A cell viability of >80%, 50–80%, and <50% is considered as non-toxic, low-toxic, and toxic, respectively. The cell viability of L929 cells treated with HACC-TPP and BIMIXHAC-TPP nanogels was above 80% at the concentration of 400 μg/mL, indicating that the HACC-TPP and BIMIXHAC-TPP nanogels were non-poisonous. Further, the cell viability of HACC-D-TPP and BIMIXHAC-D-TPP was 83.86% and 83.48% at the concentration of 50 μg/mL, respectively. At the concentration of 400 μg/mL, the cell viability of all the DOX.HCl-loaded nanogels were 71.36% and 71.48%, respectively, which is considered to be low-toxic. Overall, the cytotoxicity of HACC-D-TPP and BIMIXHAC-D-TPP nanogels was significantly weaker than that of DOX.HCl (24.13%). The biocompatibility of HACC-TPP and BIMIXHAC-TPP in this paper was preliminarily demonstrated by the L929 cells test. HACC-TPP and BIMIXHAC-TPP nanogels had good biocompatibility and non-toxicity. If further studies on the effects of nanogels on metabolism and pharmacokinetics in vivo are needed, further trials will be conducted.

## 3. Materials and Methods

### 3.1. Materials

Chitosan with a degree of deacetylation of 90% (Mw = 50 k Da,) was purchased from Golden-Shell Pharmaceutical Co., Ltd. (Zhejiang, China). The 2-Benzimidazolecarboxylic acid (BIM), isopropyl alcohol, sodium hydroxide, 2,3-epoxypropyltrimethyl ammonium chloride (EPTAC), sodium tripolyphosphate (TPP), 2,2-diphenyl-1-picrylhydrazyl (DPPH) radical, tris(hydroxymethyl)methyl aminomethane (Tris), reduced coenzyme I (NADH I), hydrochloric acid, phenazine methosulfate (PMS), and nitroblue tetrazolium (NBT) were purchased from Sinopharm Chemical Reagent Co., Ltd. (Shanghai, China). The 2% rabbit erythrocyte suspension (Sbjbio, Nanjing, China). The mouse fibroblast cell line (L929), human lung adenocarcinoma epithelial cell line (A549), and human breast cancer cell line (MCF-7) were provided by the Shanghai Institute of Biochemistry and Cell Biology, Chinese Academy of Sciences. All the reagents were analytical grade and used without further purification.

### 3.2. Synthesis of Hydroxypropyl Trimethyl Ammonium Chloride Chitosan (HACC), and HACC Grafting Benzimidazolic Acid (BIMIXHAC)

**Scheme 2:**
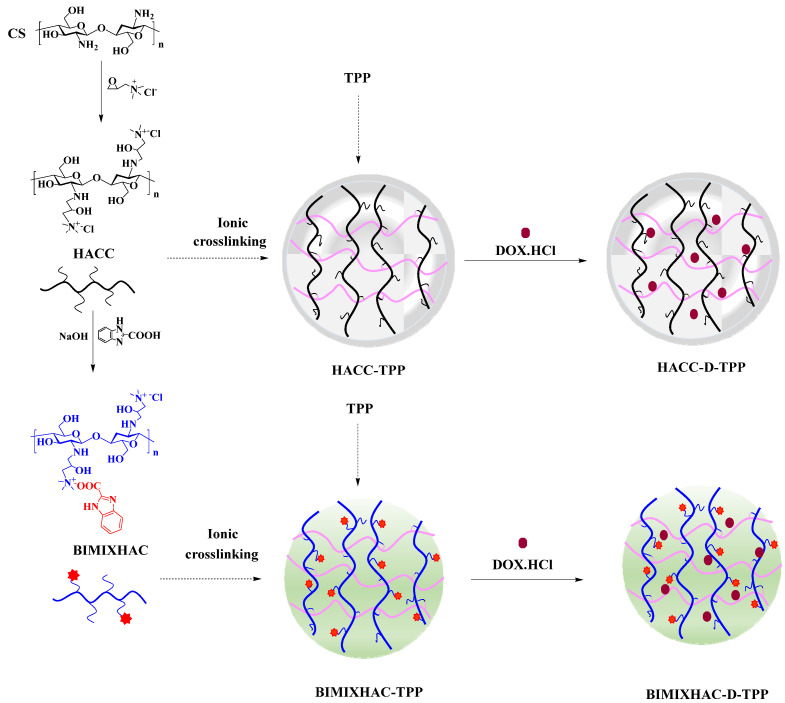
Schematic illustration of the synthesis of HACC and BIMIXHAC, the preparation of HACC-TPP, HACC-D-TPP, BIMIXHAC-TPP, and BIMIXHAC-D-TPP nanogels.

Chitosan (1.61 g, 10 mmol) was dispersed in 40 mL of isopropanol for 1.5 h at 50 °C. EPTAC (8 g, 50 mmol) was dissolved in 10 mL of of water, and the EPTAC solution was slowly dropped into chitosan solution. After the reaction was carried out for 24 h at 80 °C, the mixture was deposited in ethyl alcohol and the product of HACC was obtained. The NaOH solution of 2-benzimidazolecarboxylic acid (15 mmol) was dropped into the HACC aqueous solution and stirred for 18 h at 25 °C. Ultimately, the mixture solution was dialyzed against soft water for 3–4 days and freeze-dried for application (Scheme 2).

### 3.3. Analytical Methods

#### 3.3.1. Fourier Transform Infrared Spectroscopy (FT-IR)

The structures of all the samples were recorded using a Nicolet iS 50 Fourier Transform Infrared Spectrometer (Thermo Fisher Scientific, Waltham, MA, USA) at a wavelength range of 4000–400 cm^−1^ and a resolution of 4 cm^−1^. The samples were prepared with KBr.

#### 3.3.2. Nuclear Magnetic Resonance Spectroscopy (NMR)

All the samples were dissolved in D_2_O (4.69 ppm) with 20 mg/mL and recorded with an AVIII-500 Spectrometer (Switzerland, provided by Bruker Tech. and Serv. Co., Ltd., Beijing, China) at 500 MHz and 25 °C. The degrees of substitution (DS) were determined according to the ratio of the integral of different hydrogen protons. For instance, the DS of BIMIXHAC was measured in the following equation:

$$DS\left(\%\right)=\left[\frac{\frac{I_{He,BIMIXHAC}}{2}}{I_{H1,\;BIMIXHAC}}\right]\times 100$$

where *I_He_, _BIMIIXHAC_* represents the integral of the –CH– (He, 7.59 ppm) of BIMIXHAC, 2 represents the number of protons in the He of BIMIXHAC, and *I_H*1*, BIMIIXHAC_* represents the integral of the hydrogen atom bonded to C-1 of BIMIXHAC backbone (4.34 ppm).

### 3.4. Preparation of Nanogels Based on HACC and BIMIXHAC

As shown in Scheme 2, HACC and BIMIXHAC loading DOX.HCl nanogels were prepared by ionic crosslinking with the polyanion of TPP. The nanogels were denoted as HACC-TPP, HACC-D-TPP, BIMIXHAC-TPP, and BIMIXHAC-D-TPP, respectively. The optimum reaction condition was explored based on the zeta potential, particle size, and storage stability of nanogels. The preparation of BIMIXHAC-TPP and BIMIXHAC-D-TPP nanogels are examples for explanation. Briefly, BIMIXHAC (0.4 g) was dissolved in 100 mL of deionized water, and TPP (0.1 g) was dissolved in 50 mL of water. While stirring well, the BIMIXHAC and TPP aqueous solutions were filtered using a 0.45-micron filter for standby. The blank BIMIXHAC-TPP nanogel was prepared by adding (20 drops/min) 2.0 mL of TPP (2 mg/mL) solution into 10.0 mL of BIMIXHAC (4 mg/mL) solution, with stirring (750 rpm) for 25 min at 25 °C. The BIMIXHAC-D-TPP nanogel was prepared by dropping 2.0 mL of TPP solution containing DOX.HCl into 10.0 mL of BIMIXHAC (4 mg/mL) solution, with stirring (750 rpm) for 20 min at 25 °C. BIMIXHAC-TPP and BIMIXHAC-D-TPP were separated by centrifugation at 12,000 r/min for 30 min. The preparation of HACC-TPP and HACC-D-TPP were similar to BIMIXHAC-TPP and BIMIXHAC-D-TPP nanogels. Finally, the dried nanogels were stored for purpose.

### 3.5. Characterization of Nanogels

#### 3.5.1. Zeta Potential, Particle Size, Polydispersity Index (PDI) and Storage Stability of Nanogels

The nanoparticle size analyzer (Litesizer 500, Anton Paar Instruments, Graz, Austria) with dynamic light scattering (DLS) and electrophoretic light scattering (ELS) was used to gauge the zeta potential, particle size, and PDI three times at room temperature.

#### 3.5.2. Morphology

The morphologies of nanogels were perceived by Scanning Electron Microscopy (SEM, S-4800, Hitachi, Tokyo, Japan) and Transmission Electron Microscopy (TEM, JEM-1400 Plus, Tokyo, Japan). The nanogels for SEM and TEM analysis were prepared by dropping fresh suspension solutions on silicon wafers and copper grids, respectively.

#### 3.5.3. Drug Entrapped Efficiency (EE) and Loading Efficiency (LE) of Nanogels

The microplate reader (SuperMax, Shanghai, China) was used to detect the concentration of DOX.HCl retrieved from the solution at 480 nm. The standard curve (y = 0.0615 x − 0.011, R^2^ = 0.9936) was used to calculate the amount of DOX.HCl. The EE and LE of the DOX.HCl was determined as follows [65]:

$$EE\left(\%\right)=\left[\frac{M_{t}-M_{f}}{M_{t}}\right]\times 100$$

$$LE\left(\%\right)=\left[\frac{M_{t}-M_{f}}{M_{n}\;}\right]\times 100$$

where *M*_t_ represents the total mass of added DOX.HCl, *M*_f_ represents the mass of DOX.HCl recovered from the supernatant, and *M*_n_ represents the mass of nanogels used, respectively.

### 3.6. In Vitro Drug Release

DOX.HCl sustained-release performances of DOX.HCl-loaded nanogels (HACC-D-TPP and BIMIXHAC-D-TPP) under a series of pH conditions (pH = 5.0, 6.5, 7.4) were obtained at 480 nm. Briefly, 3 mL of DOX.HCl and DOX.HCl-loaded nanogel solutions were respectively put in dialysis bags (MW: 8000–14,000 Da) and dialyzed against in 150 mL of appropriate pH phosphate buffer with stirring at 110 rpm and 37 °C. At the scheduled time (2, 4, 6, 12, 24, 36, 48, 60, and 72 h), 3 mL of release media was collected, and then 3 mL of fresh phosphate buffer was compensated immediately. The DOX.HCl release percentages from nanogels were measured as follows [66]:

$$Cumulative\;release\left(\%\right)=\left.\frac{M_{c}}{M_{t}\;}\right.\times 100$$

where *M*_c_ and *M*_t_ represent the cumulative quantity of the DOX.HCl released at the scheduled time and the initial quality of DOX.HCl.

### 3.7. Antioxidant Assay

#### 3.7.1. DPPH Radical Scavenging Activity

All samples were prepared at a concentration of 10.0 mg/mL for standby. The sample solutions were mixed with deionized water so that the final concentrations were 0.1, 0.2, 0.4, 0.8, and 1.6 mg/mL. A total of 2.0 mL of DPPH/ethanol (180 μmol/L) was added to the mixtures. Instead of using 2.0 mL of DPPH/ethanol, 2.0 mL of ethanol was utilized as the control. The blank was prepared by the addition of 2.0 mL of distilled water along with 2.0 mL of DPPH/ethanol. Incubation of the mixtures in the dark was performed for 20 min at room temperature. The detection was performed at a wavelength of 517 nm. The DPPH radical scavenging effect was measured by the following equation [46]:

$$Scavenging\;effect\left(\%\right)=\left[1-\frac{A_{sample\;517\;nm}-A_{control\;517\;nm}}{A_{blank\;517\;nm}}\right]\times 100$$

where *A*_sample 517 nm_, *A*_control 517 nm_, and *A*_blank 517 nm_ represent the absorbance of the sample, the control, and the blank.

#### 3.7.2. Superoxide Anion Radical Scavenging Activity

All samples were prepared at a concentration of 10.0 mg/mL for standby. All the sample solutions were mixed with water to a volume of 1.5 mL. Subsequently, 0.5 mL of NADH (0.2453 mg/mL), 0.5 mL of NBT (0.3657 mg/mL), and 0.5 mL of PMS (0.01838 mg/mL) were added into sample solutions. The mixture solutions were kept in the dark for 5 min. The detection was conducted at a wavelength of 560 nm. The control solutions were provided by substituting NADH with Tris-HCl buffer (1.9382 mg/mL, pH 8.0). The blank, used to substitute the sample solutions, consisted of Tris-HCl buffer (pH 8.0). The calculation of the superoxide anion radical scavenging effect was carried out using the following equation [46]:

$$Scavenging\;effect\;(\%)=\left[1-\frac{A_{sample\;560\;nm}-A_{control\;560\;nm}}{A_{blank\;560\;nm}}\right]\times 100$$

where *A*_sample 560 nm_, *A*_control 560 nm_, and *A*_blank 560 nm_ represent the absorbance of the sample, the control, and the blank.

### 3.8. Hemocompatibility Assay

0.5 mL of blank nanogels (0.5, 1.0, 1.5, and 2.0 mg/mL) were mixed with 0.5 mL of the 2% rabbit erythrocyte suspension. After incubating in a water bath at 37 °C for 6, 12, and 24 h, the mixtures were centrifuged at 3000 rpm for 5 min, and the obtained supernatant was analyzed using a microplate reader at 545 nm. The phosphate buffer (pH 7.4) and double distilled water were used as negative control and positive control. The hemolytic ratio was identified utilizing the following formula [65]:

$$Hemolytic\;ratio\;(\%)=\left[\frac{A_{sample}-A_{negative}}{A_{positive}-A_{negative}}\right]\times 100$$

where *A_sample_* represents the 2% erythrocytes that were incubated with the sample, *A_negative_* represents the phosphate buffer solution (pH 7.4), and *A_positive_* represents double distilled water.

### 3.9. Cell Cytotoxicity Assay

The MTT assay was used to assess the cell cytotoxicity of all the nanogels on A549, MCF-7, and L929 cells. Briefly, all the cells were cultured on 96-well plates (10 × 10^4~5^ cells/per well). The A549, MCF-7, and L929 cells were treated with different concentrations (400, 200, 100, 50, and 25 μg/mL). After incubation in a cell incubator for 48 h, 100 μL of MTT salt solution (0.5 mg/mL) was added to each well and incubated for 4 h. To separate the MTT solution, 100 µL of DMSO was added to each well with a 15 min stopover. The absorbance was determined at 490 nm. The cell viability was calculated utilizing the following equation [67]:

$$Cellv\;iability\;(\%)=\left.\frac{A_{sample}}{A_{blank}}\right.\times 100$$

where *A*_sample_ represents the absorbance value of the sample, and *A*_blank_ represents the absorbance of the blank.

### 3.10. Statistical Analysis

The statistical analysis involved using a Student’s test and a one-way analysis of variance in IBM SPSS 22. All the tests were carried out in triplicate and the results were presented as the mean ± standard deviation (SD). Significance was determined for data with a *p*-value less than 0.05.

## 4. Conclusions

In this study, chitosan nanogels loading DOX.HCl based on BIMIXHAC was successfully designed as a nanocarrier with enhanced antioxidant activity, antitumor activity, and reduced cytotoxicity. The correct structures of all the nanogels (HACC-TPP, HACC-D-TPP, BIMIXHAC-TPP, and BIMIXHAC-D-TPP) were verified by FT-IR, ELS, and DLS. The surface morphology of all the nanogels observed by SEM and TEM was oval. The nanogels showed a uniform particle size (242.78 ± 5.31 nm), positive zeta potential (+20.12 ± 0.36 mV), and a relatively small value of PDI (27.67 ± 0.44%). The stability of the nanogels showed that the particle size and zeta potential of the nanogels did not change much with the passage of time, indicating that nanogels were relatively stable. The EE and LE of BIMIXHAC-D-TPP nanogels were estimated, which were 93.17 ± 0.27% and 31.17 ± 0.09%, respectively. The DOX.HCl release from the nanogels exhibited an excellent response to pH, with the quickest release at a pH of 5.0 in phosphate buffer solution. The results showed that the nanogel had a stable pH sensitivity in the release medium and could be used as a controllable nanocarrier for DOX.HCl. Meanwhile, the antioxidant capacity of the nanogels was assayed, and the nanogels displayed available enhancement of radical scavenging activity. The hemolysis assay displayed a good blood compatibility of the blank nanogels of BIMIXHAC-TPP and HACC-TPP. The assay of cell viability by the MTT test was proven, and the results showed that both the BIMIXHAC-D-TPP and HACC-D-TPP nanogels possessed strong antitumor activities. Further, the antitumor activity against the cancer cells of BIMIXHAC-D-TPP was much higher than HACC-D-TPP, which might relate to the bioactivity of benzimidazole acid. Additionally, to evaluate the biocompatibility of the DOX.HCl-loaded nanogels, the cytotoxicity against normal L929 cells was investigated. The results showed that there was a remarkable increase in cell viability at different concentrations after DOX.HCl loading on nanogels. The results showed that the nanogel based on BIMIXHAC had good biocompatibility, enhanced bioactivity, and pH-responsive release, and could be a promising nanocarrier. The findings of this study suggest that benzimidazole-chitosan quaternary ammonium salt nanogels hold promise as a nanocarrier. Nevertheless, to ascertain the complete drug delivery capability of the benzimidazole-chitosan quaternary ammonium salt nanogel carrier, further extensive research, including in vivo experiments, will be carried out in the subsequent phase of this study.

## Figures and Tables

**Table 1:** Zeta potential, particle size, PDI, EE, and LE of all the nanogels.

Sample	Size (nm)	Zeta Potential (mV)	PDI (%)	EE (%)	LE (%)
HACC-TPP	132.21 ± 4.51	18.12 ± 0.06	28.18 ± 1.09	–	–
HACC-D-TPP	199.6 ± 4.87	20.69 ± 0.27	28.25 ± 0.91	90.02 ± 0.25	26.74 ± 0.07
BIMIXHAC-TPP	172.33 ± 4.95	20.97 ± 0.17	26.39 ± 0.51	–	–
BIMIXHAC-D-TPP	242.78 ± 5.31	20.12 ± 0.36	27.67 ± 0.44	93.17 ± 0.27	31.17 ± 0.09

## Data Availability

All data are contained in the manuscript.

## References

[1] Huang J.L., Song S.S., Wang M., Wang H.X. (2023). Transforming a highly toxic agent DM1 into injectable safe nanomedicines via prodrug self-assembly for the treatment of taxane-resistant cancer. Nanoscale.

[2] Oroudjev E., Lopus M., Wilson L., Audette C., Provenzano C., Erickson H., Kovtun Y., Chari R., Jordan M.A. (2010). Maytansinoid-antibody conjugates induce mitotic arrest by suppressing microtubule dynamic instability. Mol. Cancer Ther..

[3] Li W.J., Li S.H., Xu G., Man X.Y., Yang T.F., Zhang Z.L., Liang H., Yang F. (2023). Developing a Ruthenium(III) Complex to Trigger Gasdermin E-Mediated Pyroptosis and an Immune Response Based on Decitabine and Liposomes: Targeting Inhibition of Gastric Tumor Growth and Metastasis. J. Med. Chem..

[4] Christowitz C., Davis T., Isaacs A., van Niekerk G., Hattingh S., Engelbrecht A.M. (2019). Mechanisms of doxorubicin-induced drug resistance and drug resistant tumour growth in a murine breast tumour model. BMC Cancer.

[5] Hofmann W.K., Trumpp A., Müller-Tidow C. (2023). Therapy resistance mechanisms in hematological malignancies. Int. J. Cancer.

[6] Wang J., Wang F.L., Xie D.D., Zhou M., Liao J.X., Wu H.L., Dai Y., Huang J.B., Zhao Y. (2023). PLGA Nanoparticles Containing VCAM-1 Inhibitor Succinobucol and Chemotherapeutic Doxorubicin as Therapy against Primary Tumors and Their Lung Metastases. Pharmaceutics.

[7] Karimi-Soflou R., Karkhaneh A. (2022). Redox-Sensitive multifunctional hyaluronic acid-based nanomicelles with Fine-controlled anticancer drug release. Int. J. Pharm..

[8] Renu K., Abilash V.G., Pichiah P.B.T., Arunachalam S. (2018). Molecular mechanism of doxorubicin-induced cardiomyopathy–An update. Eur. J. Pharmacol..

[9] Singh M., Nicol A.T., DelPozzo J., Wei J., Singh M., Nguyen T. (2022). Demystifying the relationship between metformin, AMPK, and doxorubicin cardiotoxicity. Front. Cardiovasc. Med..

[10] Danan G., Teschke R. (2015). RUCAM in Drug and Herb Induced Liver Injury: The Update. Int. J. Mol. Sci..

[11] Kassner N., Huse K., Martin H.J., Godtel-Armbrust U., Metzger A., Meineke I., Brockmoller J., Klein K., Zanger U.M., Maser E. (2008). Carbonyl reductase 1 is a predominant doxorubicin reductase in the human liver. Drug Metab. Dispos..

[12] Lahoti T.S., Patel D., Thekkemadom V., Beckett R., Ray S.D. (2012). Doxorubicin-induced in vivo nephrotoxicity involves oxidative stress-mediated multiple pro- and anti-apoptotic signaling pathways. Curr. Neurovasc. Res..

[13] Mei S.B., Hong L., Cai X.Y., Xiao B., Zhang P., Shao L. (2019). Oxidative stress injury in doxorubicin-induced cardiotoxicity. Toxicol. Lett..

[14] Li D.L., Hill J.A. (2014). Cardiomyocyte autophagy and cancer chemotherapy. J. Mol. Cell. Cardiol..

[15] Sun J., Zhou J.D., Sun S.M., Lin H., Zhang H.L., Zhong Z.Q., Chi J.F., Guo H.Y. (2023). Protective effect of urotensin II receptor antagonist urantide and exercise training on doxorubicin-induced cardiotoxicity. Sci. Rep..

[16] Hashimoto Y., Mukai S.A., Sasaki Y., Akiyoshi K. (2018). Nanogel Tectonics for Tissue Engineering: Protein Delivery Systems with Nanogel Chaperones. Adv. Healthc. Mater..

[17] Sankaranarayanan A., Ramprasad A., Ganesh S.S., Ganesh H., Ramanathan B., Shanmugavadivu A., Selvamurugan N. (2023). Nanogels for bone tissue engineering-from synthesis to application. Nanoscale.

[18] Eckmann D.M., Composto R.J., Tsourkas A., Muzykantov V.R. (2014). Nanogel carrier design for targeted drug delivery. J. Mater. Chem. B.

[19] Geyik G., Guncum E., Isiklan N. (2023). Design and development of pH-responsive alginate-based nanogel carriers for etoposide delivery. Int. J. Biol. Macromol..

[20] Dimatteo R., Darling N.J., Segura T. (2018). In situ forming injectable hydrogels for drug delivery and wound repair. Adv. Drug Deliv. Rev..

[21] Duan Q.Y., Zhu Y.X., Jia H.R., Wang S.H., Wu F.G. (2023). Nanogels: Synthesis, properties, and recent biomedical applications. Prog. Mater. Sci..

[22] Soni G., Yadav K.S. (2016). Nanogels as potential nanomedicine carrier for treatment of cancer: A mini review of the state of the art. Saudi Pharm. J..

[23] Cuenot S., Radji S., Alem H., Demoustier-Champagne S., Jonas A.M. (2012). Control of swelling of responsive nanogels by nanoconfinement. Small.

[24] Quan F.Y., Zhang A.T., Cheng F.F., Cui L., Liu J.Q., Xia Y.Z. (2018). Biodegradable polymeric architectures via reversible deactivation radical polymerizations. Polymers.

[25] Soni K.S., Desale S.S., Bronich T.K. (2016). Nanogels: An overview of properties, biomedical applications and obstacles to clinical translation. J. Control. Release.

[26] Altuntas E., Özkan B., Güngör S., Özsoy Y. (2023). Biopolymer-Based Nanogel Approach in Drug Delivery: Basic Concept and Current Developments. Pharmaceutics.

[27] Li H.P., Zhou Z.W., Zhang F.R., Guo Y.X., Yang X., Jiang H.L. (2018). A networked swellable dextrin nanogels loading Bcl2 siRNA for melanoma tumor therapy. Nano Res..

[28] Goldberg M., Langer R., Jia X.Q. (2007). Nanostructured materials for applications in drug delivery and tissue engineering. J. Biomater. Sci. Polym. Ed..

[29] Myerson J.W., McPherson O., DeFrates K.G., Towslee J.H., Marcos-Contreras O.A., Shuvaev V.V., Braender B., Composto R.J., Muzykantov V.R., Eckmann D.M. (2019). Cross-linker-Modulated Nanogel Flexibility Correlates with Tunable Targeting to a Sterically Impeded Endothelial Marker. ACS Nano.

[30] Zhang Y.Y., Wang T.G., Zhuang Y.P., He T.D., Wu X.L., Su L., Kang J., Chang J., Wang H.J. (2021). Sodium Alginate Hydrogel-Mediated Cancer Immunotherapy for Postoperative in Situ Recurrence and Metastasis. ACS Biomater. Sci. Eng..

[31] Hu Y., Zhao M., Wang H., Guo Y., Cheng X.L., Zhao T., Wang H.Q., Zhang Y.F., Ma Y., Tao W.W. (2023). Exosome-sheathed ROS-responsive nanogel to improve targeted therapy in perimenopausal depression. J. Nanobiotechnol..

[32] Yang H.Y., Jang M.S., Sun X.S., Liu C.L., Lee J.H., Li Y., Fu Y. (2023). CD44-mediated tumor homing of hyaluronic acid nanogels for hypoxia-activated photodynamic therapy against tumor. Colloid Surf. B.

[33] Cong X.F., Chen J., Xu R. (2022). Recent Progress in Bio-Responsive Drug Delivery Systems for Tumor Therapy. Front. Bioeng. Biotechnol..

[34] Faubert B., Solmonson A., DeBerardinis R.J. (2020). Metabolic reprogramming and cancer progression. Science.

[35] Heawchaiyaphum C., Yoshiyama H., Iizasa H., Burassakarn A., Tumurgan Z., Ekalaksananan T., Pientong C. (2023). Epstein-Barr Virus Promotes Oral Squamous Cell Carcinoma Stemness through the Warburg Effect. Int. J. Mol. Sci..

[36] Flavahan W.A., Wu Q., Hitomi M., Rahim N., Kim Y., Sloan A.E., Weil R.J., Nakano I., Sarkaria J.N., Stringer B.W. (2013). Brain tumor initiating cells adapt to restricted nutrition through preferential glucose uptake. Nat. Neurosci..

[37] Raines L.N., Huang S.C. (2021). Is glucose the scapegoat for tumor evasion. Cancer Cell..

[38] Bhaladhare S., Bhattacharjee S. (2023). Chemical, physical, and biological stimuli-responsive nanogels for biomedical applications (mechanisms, concepts, and advancements): A review. Int. J. Biol. Macromol..

[39] Cinay G.E., Erkoc P., Alipour M., Hashimoto Y., Sasaki Y., Akiyosh K., Kizilel S. (2017). Nanogel-Integrated pH-Responsive Composite Hydrogels for Controlled Drug Delivery. ACS Biomater. Sci. Eng..

[40] Pragti B.K., Kundu B.K., Singh S., Ranjith W.A.C., Sarkar S., Sonawane A., Mukhopadhyay S. (2023). Chitosan-Biotin-Conjugated pH-Responsive Ru(II) Glucose Nanogel: A Dual Pathway of Targeting Cancer Cells and Self-Drug Delivery. ACS Appl. Mater. Interfaces.

[41] Mahmoodzadeh F., Ghorbani M., Jannat B. (2019). Glutathione and pH-responsive chitosan-based nanogel as an efficient nanoplatform for controlled delivery of doxorubicin. J. Drug Deliv. Sci. Technol..

[42] Li D.Y., Xu W.S., Liu H. (2023). Fabrication of chitosan functionalized dual stimuli-responsive injectable nanogel to control delivery of doxorubicin. Colloid Polym. Sci..

[43] Lakkakula J.R., Gujarathi P., Pansare P., Tripathi S. (2021). A comprehensive review on alginate-based delivery systems for the delivery of chemotherapeutic agent: Doxorubicin. Carbohydr. Polym..

[44] Meng Q.Y., Zhong S.L., Xu L.F., Wang J.F., Zhang Z.Q., Gao Y., Cui X.J. (2022). Review on design strategies and considerations of polysaccharide-based smart drug delivery systems for cancer therapy. Carbohydr. Polym..

[45] Tabriz A., Alvi M.A.U.R., Niazi M.B.K., Batool M., Bhatti M.F., Khan A.L., Khan A.U., Jamil T., Ahmad N.M. (2019). Quaternized trimethyl functionalized chitosan based antifungal membranes for drinking water treatment. Carbohydr. Polym..

[46] Ma B., Zhang J.J., Mi Y.Q., Miao Q., Tan W.Q., Guo Z.Y. (2023). Preparation of imidazole acids grafted chitosan with enhanced antioxidant, antibacterial and antitumor activities. Carbohydr. Polym..

[47] Mi Y.Q., Zhang J.J., Zhang L.L., Li Q., Cheng Y.Z., Guo Z.Y. (2021). Synthesis, Characterization, and Evaluation of Nanoparticles Loading Adriamycin Based on 2-Hydroxypropyltrimethyl Ammonium Chloride Chitosan Grafting Folic Acid. Polymers.

[48] Ma B., Tan W.Q., Zhang J.J., Mi Y.Q., Miao Q., Guo Z.Y. (2023). Preparation and Characterization of Chitosan Derivatives Bearing Imidazole Ring with Antioxidant, Antibacterial, and Antifungal Activities. Starch-Stärke.

[49] Pan Q.Y., Zhou C., Yang Z.M., He Z.Y., Wang C., Liu Y.H., Song S.H., Gu H., Hong K.Q., Yu L.J. (2022). Preparation and characterization of chitosan derivatives modified with quaternary ammonium salt and quaternary phosphate salt and its effect on tropical fruit preservation. Food Chem..

[50] Tang F.L., Lv L.M., Lu F., Rong B., Li Z.Q., Lu B.T., Yu K., Liu J.W., Dai F.Y., Wu D.Y. (2016). Preparation and characterization of *N*-chitosan as a wound healing accelerator. Int. J. Biol. Macromol..

[51] Wang F., Zhang Q., Huang K.X., Li J.R., Wang K., Zhang K., Tang X.Y. (2020). Preparation and characterization of carboxymethyl cellulose containing quaternized chitosan for potential drug carrier. Int. J. Biol. Macromol..

[52] Mendapara J.V., Vaghasiya M.D., Rajani D.P., Ahmad I., Patel H., Kumari P. (2023). Benzimidazole and piperidine containing novel 1,2,3-triazole hybrids as anti-infective agents: Design, synthesis, in silico and in vitro antimicrobial efficacy. J. Biochem. Mol. Toxicol..

[53] Zafar S., Ashraf S., Iqbal U., Yousuf S., Siddiqi H.M., Liaqat F., Rehman H.M., Akhtar Z., El-Shafei A., Shahzad N. (2023). Synthesis of new benzimidazole based ruthenium (II) dyes for application in dye-sensitized solar cells with detailed spectroscopic and theoretical evaluation. J. Mol. Struct..

[54] Wang S., Pi L., Wen H., Yu H., Yang X. (2020). Evaluation of novel magnetic targeting microspheres loading adriamycin based on carboxymethyl chitosan. J. Drug Deliv. Sci. Technol..

[55] Xu X.F., Li Y.G., Wang F.H., Lv L., Liu J.Y., Li M.N., Guo A.J., Jiang J.J., Shen Y.Y., Guo S.R. (2013). Synthesis, in vitro and in vivo evaluation of new norcantharidin-conjugated hydroxypropyltrimethyl ammonium chloride chitosan derivatives as polymer therapeutics. Int. J. Pharm..

[56] Mahalingam S., Murugesan A., Thiruppathiraja T., Lakshmipathi S., Makhanya T.R., Gengan R.M. (2022). Green synthesis of benzimidazole derivatives by using zinc boron nitride catalyst and their application from DFT (B3LYP) study. Heliyon.

[57] Xiang S.D., Scholzen A., Minigo G., David C., Apostolopoulos V., Mottram P.L., Plebanski M. (2006). Pathogen recognition and development of particulate vaccines: Does size matter. Methods.

[58] Zhou G.Z., Gao Y., Shi Y.B., Qiu S.N., Lin G.M., Ding X.B., Wang W.G., Feng Y.H., Wang F., Qiao J.W. (2024). Design of in vitro biomimetic experimental system and simulation analysis for transvascular transport of nano-preparation. Microvas. Res..

[59] Panda P.K., Jain S.K. (2023). Doxorubicin bearing peptide anchored PEGylated PLGA nanoparticles for the effective delivery to prostate cancer cells. J. Drug Deliv. Sci. Technol..

[60] Kelishomi R.B., Ejtemaeemehr S., Tavangar S.M., Rahimian R., Mobarakeh J.I., Dehpour A.R. (2008). Morphine is protective against doxorubicin-induced cardiotoxicity in rat. Toxicology.

[61] Rahimi_Balaei M., Momeny M., Babaeikelishomi R., Mehr S.E., Tavangar S.M., Dehpour A.R. (2010). The modulatory effect of lithium on doxorubicin-induced cardiotoxicity in rat. Eur. J. Pharmacol..

[62] Khalilzadeh M., Abdollahi A., Abdolahi F., Abdolghaffari A.H., Dehpour A.R., Jazaeri F. (2018). Protective effects of magnesium sulfate against doxorubicin induced cardiotoxicity in rats. Life Sci..

[63] Zhang T.Y., Li N.N., Wang R., Sun Y.Y., He X.Y., Lu X.Y., Chu L.X., Sun K.X. (2023). Enhanced therapeutic efficacy of doxorubicin against multidrug-resistant breast cancer with reduced cardiotoxicity. Drug Deliv..

[64] Wang G.L., Fan C., Wang H., Jia C.Y., Li X.T., Yang J.R., Zhang T., Gao S., Min X., Huang J. (2022). Type VI secretion system-associated FHA domain protein TagH regulates the hemolytic activity and virulence of Vibrio cholera. Gut Microbes.

[65] Qiu J.G., Tomeh M.A., Jin Y., Zhang B., Zhao X.B. (2023). Microfluidic formulation of anticancer peptide loaded ZIF-8 nanoparticles for the treatment of breast cancer. J. Colloid Interf. Sci..

[66] Mi Y.Q., Zhang J.J., Tan W.Q., Miao Q., Li Q., Guo Z.Y. (2022). Preparation of Doxorubicin-Loaded Carboxymethyl-β-Cyclodextrin/Chitosan Nanoparticles with Antioxidant, Antitumor Activities and pH-Sensitive Release. Mar. Drugs.

[67] Ma B., Li Q., Mi Y.Q., Zhang J.J., Tan W.Q., Guo Z.Y. (2023). pH-responsive nanogels with enhanced antioxidant and antitumor activities on drug delivery and smart drug release. Int. J. Biol. Macromol..

